# Sorafenib in patients with progressed and refractory bone tumors

**DOI:** 10.1007/s12032-018-1180-x

**Published:** 2018-08-16

**Authors:** Anna Raciborska, Katarzyna Bilska

**Affiliations:** Department of Oncology and Surgical Oncology for Children and Youth, Institute of Mother and Child, 01-211 Warszawa, ul. Kasprzaka 17a, Warsaw, Poland

**Keywords:** Sorafenib, Osteosarcoma, Ewing sarcoma, Chondrosarcoma, Children, Young adults

## Abstract

Patients with metastatic, progressive, or recurrent bone tumors have a dismal outcome. Sorafenib has been proposed as an effective salvage regimen for some malignancies. Thus, we sought to evaluate this approach for young patients with relapsed or refractory bone tumors. Twelve patients with refractory bone tumors (two with Ewing sarcoma, two with chondrosarcoma, and eight with osteosarcoma) received salvage treatment with sorafenib. All patients had standard tumor imaging and laboratory evaluation. All toxicities were documented. At the time of the beginning of sorafenib treatment median age among 12 patients was 18 years (range 4.1–27.9 years), eight were male, and eight had osteosarcoma. All received sorafenib because of relapse. Seven patients were treated parallel to other standard chemotherapy. Overall response rate was 75%. Median time to sorafenib time to progression for patients with osteosarcoma was 4 months (range 1.8–7.9 months). Four patients (33%) are alive, in that two with no evidence of disease with a median follow-up of 41 months (range 26.5–60.9 months). The estimated 5 year overall survival (OS) for the whole group was 64.49%. There were no serious toxicities. Sorafenib is well-tolerated in young patients with bone tumors, and particularly could be an option for patients with metastatic disease and refractory osteosarcoma. Sorafenib only allows to extend OS and different procedures are needed to achieve permanent remission. This regimen deserves further investigation in the upfront management of patients with high-risk bone tumors.

## Introduction

Approximately 1100–1200 new cases of malignant neoplasm in children are diagnosed in Poland every year. Out of that group all bone tumors consist about 7%. The frequency of occurrence increases with age. Adolescents and young adults are the most common group of patients, although appearance of such diseases is also not rare among the children’s early age group. The most commonly diagnosed bone cancers are osteosarcoma (OS) and Ewing sarcoma (ES)—56% and 34%, respectively. With advances in multimodal therapy, survival rates for patients with primary localized bone disease approaches 65–75% [1, 2]. However, patients with metastatic, progressive, or recurrent diseases have a dismal outcome [1–5].

For some malignancies sorafenib has been proposed as an effective drug, particularly primary kidney cancer, liver cancer, and thyroid carcinoma. Furthermore, it has been proposed as an oral agent in the therapy of high grade progressing osteosarcoma in adult patients and in refractory solid tumors in children and young adults [6–11]. Sorafenib is a kinase inhibitor drug and is available as an oral formulation; compared to other drugs from the same group sorafenib inhibits also Raf, Mek, and Erk kinase pathways. Both the moderate toxicity profile and the promising results observed in a few studies allow to consider sorafenib as a reasonable treatment option also for heavily pretreated young patients with bone tumors [7, 8, 12]. Thus, we sought to evaluate this schedule, particularly the response rate and progression-free survival for patients with refractory or relapsed bone tumors.

## Materials and methods

### Patients

Twelve patients with histologically confirmed primary bone tumors were treated with sorafenib during the period 2015–2017 at the Mother and Child Institute (Warsaw, Poland). Prior to treatment informed consent was obtained from all patients. In cases when minors were involved the consent was obtained from their legal guardians. Approval for this retrospective study was obtained in compliance with the international regulations for protection of human research subjects (Bio-ethical Committee at the Mother and Child Institute in Warsaw, opinion issued under number 35/2018).

### Treatment

Sorafenib was administered at a dose of 400 mg twice a day in patients older than 15 years and/or heavier than 50 kg. In younger patients the dose was calculated in proportion to their body weight and was 100 mg twice a day for patients weighing 15–20 kg, and 200 mg twice a day if the weight was 20–30 kg. Treatment was to be continued until disease progression or unacceptable toxicity. Dose reduction was undertaken in case of CTCAE v. 4.0 grades 3 and 4. Patients developing allergic symptoms received steroids and antihistaminic drugs. Patients developing leucopoenia or thrombocytopenia were treated symptomatically with G-CSF and transfusion of blood products.

### Assessment of Response and Toxicity

All patients had standard tumor imaging using CT, MRI, bone scan, or PET, as indicated, prior to starting sorafenib and every 3 months afterwards. Physical examination and laboratory evaluation were performed prior to each cycle of standard chemotherapy, every month, or weekly when necessary. All toxicities were documented from day 1 of the first day of sorafenib until end of therapy. WHO criteria were used to evaluate the response.

### Statistical methods

Overall survival (OS) was defined as the time interval from the date of diagnosis to the date of death or to last follow-up date. Time to the first relapse was defined as the time interval from date of initial biopsy to the date of the first day of the first relapse’s treatment. Sorafenib OS was defined as the time interval from the first day of sorafenib treatment to the date of death or to the last follow-up date. Sorafenib time to progression (TTP) was defined as the time interval from the start date of sorafenib to the date of disease progression. Response rate was defined as the percentage of patients who achieved stable disease (SD), partial response (PR), and complete remission (CR) during sorafenib treatment. Results’ distribution was estimated using the Kaplan–Meier method. Log-rank test was used to compare groups.

*P* ≤ 0.05 was regarded as significant. Statistical analysis was performed using STATA 10.0 for Windows.

## Results

### Patients

Between 2015 and 2017, 12 patients (8 males, 4 females) with histologically confirmed primary bone tumors (eight with osteosarcoma, two with Ewing sarcoma, and three with chondrosarcoma) were treated with oral sorafenib. Patients’ clinical characteristics are shown in Table 1. Median age at the time of diagnosis was 13.4 years (range 2.6–19.9 years). Ten patients had metastatic disease at diagnosis (seven of them only to the lungs, one of them to the bones, and two of them to the lungs and bones). Median time to the 1st relapse was 16.5 months (range 1.2–41.8 months). Sites of relapse were as follows: isolated lung metastases 6 patients (50%), isolated bone metastases 1 patient (8.3%), combined lung and bones 3 patients (25%), and local with lung metastases 2 patients (16.7%).

**Table 1:** Patient characteristics, response to sorafenib, and outcome

Pts. nb.	Age at diagn. (years)	Type of can.	Sex	Stage at diagn.	Nb. sorafenib line therapy	CHT parallel to sorafenib	Age at sorafenib	Length of therapy (days)	Best response	Time to the best response	Toxicity (grades 2–3)	Reason for stopping therapy	Status at last follow up
1	2.6	ES	F	Met	1	IFO, TC	4.1	40	PD	–	–	PD	AWD
2	15.7	CHS	M	Met	4	–	20.8	56	PR	30	–	PD	DOD
3	19.9	OS	F	Met	1	VP, IFO	20.5	147	PR	26	Skin	Toxicity	AWD
4	14.5	OS	F	Loc	2	–	21.3	299	PR	66	–	PD	DOD
5	11.0	OS	F	Loc	6	ADM, GMZ	19.6	214	PR	80	–	PD	DOD
6	18.4	OS	M	Met	5	–	27.9	71	PR	35	–	PD	DOD
7	6.7	OS	M	Met	3	DTIC,VP, CTX	7.6	114	PR	41	–	PD	DOD
8	3.5	CHS	M	Met	2	VP, CBDCA	4.1	60	PD	–	–	PD	DOD
9	13.6	OS	M	Met	2	GMZ, DCT	16.2	15	SD	15	Skin	Toxicity	NED
10	18.5	ES	M	Met	2	–	21.9	50	PR	32	–	PD	DOD
11	13.3	OS	M	Met	1	–	16.3	13	SD	13	Skin	Toxicity	NED
12	12.8	OS	M	Met	1	VP, IFO	13.0	118	PR	69	–	Surgery	DOD

*Nb* number, *ES* Ewing sarcoma, *OS* osteosarcoma, *CHS* chondrosarcoma, *Met* metastases, *Loc* localize, *TC* topotecan, cyclophosphamide, *IFO* ifosfamide, *GMZ* gemcitabine, *DCT* docetaxel, *CTX* cyclophosphamide, *VP* etoposide, *ADM* adriamycin, *DTIC* dacarbazine, *CBDCA* carboplatin, *PR* partial response, *SD* stable disease, *PD* progression disease, *DOD* death of disease, *NED* no evidence of disease, *AWD* alive with disease

### Treatment

Sorafenib was used in 5 patients as 1st, in 3 as 2nd, in 1 as 3rd, in 1 as 4th, in 1 as 5th, and in 1 as 6th line of relapse’s treatment. Median age at the start of sorafenib treatment was 18 years (range 4.1–27.9 years). Median time from the initial diagnosis to the start of sorafenib was 33.8 months (range 1.9–113.4 months). Median time of sorafenib treatment was 65.5 days (range 15–299 days). Seven patients were treated parallel to other standard chemotherapy (Table 1).

### Outcome and toxicity

**Fig. 1:**
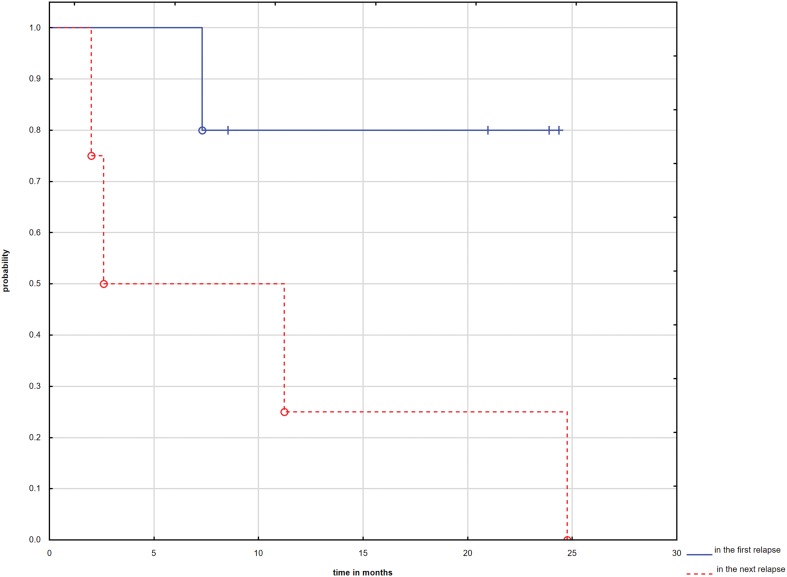
Comparison of OS between patients treated with sorafenib when it was used in the first recurrence vs. when it was used in the next recurrence

Median follow-up from start of sorafenib was 9.6 months (range 2–24.8 months). Partial response was achieved in eight patients (six with osteosarcoma), SD in two patients (both with osteosarcoma), and PD in two pts, with an overall response rate of 75%. Median time to sorafenib TTP for the whole group was 2.2 months (range 1.3–7.9 months). Median time to sorafenib TTP for the patients with osteosarcoma was 4 months (range 1.8–7.9 months). Four patients (33%) are alive including two with no evidence of disease with a median follow-up of 41 months (range 26.5–60.9 months) and median follow-up from start of sorafenib was 22.1 months (range 8.4–24 months). 5-years OS estimate for the whole group was 64.49%. The efficiency of sorafenib appears to be higher when it is used in the first recurrence, however, p is not significant (p = 0.125) (Fig. 1). Three patients had documented grade 3 skin toxicity and for that reason treatment was discontinued. There were no other significant toxicities.

## Discussion

The dismal prognosis of recurrent or progressive bone tumors highlights the importance of the development of new treatments. Over the last years, several drugs and combinations have been explored in this setting, but enough satisfactory results have not been achieved [13, 14].

Here we have presented our results with the use of sorafenib in the management of progressive or recurrent bone tumors. Importantly, there is a very small number of reported studies using sorafenib in young patients with that kind of tumors. In our group, we had four patients under the age of 15, and the youngest patient on the day of starting sorafenib treatment was only 4 years old. To our knowledge, this is the youngest patient with bone tumor who has used this type of therapy.

Some studies have described the efficacy of sorafenib in refractory bone tumor. A moderately successful response was reported in Coventon’s study regarding the application of sorafenib in advanced, relapsed, and refractory to standard chemotherapy osteosarcoma in 35 patients over 14-years-old; in 46% patients 4 months progression-free survival was observed, and 29% pts had SD over 6 months. The other study described by Coventon included 4 patients; 3 of them had SD over 3 months. Our study contained eight patients with osteosarcoma. Four of them achieved PR, two had SD. Median time to sorafenib TTP for patients with osteosarcoma was 4 months (range 1.8–7.9 months) similarly to Conventon’s study.

Preclinical data have shown the possibility of better results in combination of sorafenib with other drugs, included mTOR inhibitors [15]. Grignani has shown a study, where overall-response rate was 10% in sorafenib and everolimus combination in unresectable, advanced osteosarcoma patients older than 17 years. Unfortunately, clinical data available in the literature are still not satisfactory.

According to Saletta’s study in an early pediatric clinical trial sorafenib has shown 30% response in solid tumors and 75% in AML. A high response rate (75%) was noted in our group. Most likely the difference derives from the fact that our group was less heterogenic and included only patients with bone tumors, in majority osteosarcoma.We have not observed serious toxicity in our group (this is compatible with other different studies) although, those skin adverse events were not acceptable to our patients and their families, and they were the reason for treatment’s discontinuation.

Unfortunately, in our study the median treatment’s response for the whole group was 2.2 months. Therefore, it seems that other additional procedures (like surgery), new drugs, or strategies are necessary to achieve permanent remission in this type of patients, and at this moment sorafenib allows only to extend OS. Our study is limited and can be bias because of the small heterogeneous group and retrospective data. Nevertheless, our study confirms that sorafenib is well-tolerated in young patients with bone tumors and particularly could be an option for patients with metastatic disease and refractory osteosarcoma. Further prospective studies are needed to better define the use of sorafenib in the upfront management of these groups of patients.
